# Creation of a library of induced pluripotent stem cells from Parkinsonian patients

**DOI:** 10.1038/npjparkd.2016.9

**Published:** 2016-06-02

**Authors:** Staffan Holmqvist, Šárka Lehtonen, Margarita Chumarina, Katja A Puttonen, Carla Azevedo, Olga Lebedeva, Marika Ruponen, Minna Oksanen, Mehdi Djelloul, Anna Collin, Stefano Goldwurm, Morten Meyer, Maria Lagarkova, Sergei Kiselev, Jari Koistinaho, Laurent Roybon

**Affiliations:** 1 Stem Cell Laboratory for CNS Disease Modeling, Wallenberg Neuroscience Center, Department of Experimental Medical Science, BMC A10, Lund University, Lund, Sweden; 2 Strategic Research Area MultiPark, Lund University, Lund, Sweden; 3 Lund Stem Cell Center, Lund University, Lund, Sweden; 4 Stem Cell Laboratory of Molecular Brain Research Group, Department of Neurobiology, A.I. Virtanen Institute, University of Eastern Finland, Kuopio, Finland; 5 Russian Academy of Sciences, Vavilov Institute of General Genetics, Moscow, Russia; 6 Department of Clinical Genetics and Biobanks, Office for Medical Services, Division of Laboratory Medicine, Lund, Sweden; 7 Parkinson Institute, Istituti Clinici di Perfezionamento, Milan, Italy; 8 Department of Neurobiology Research, Institute of Molecular Medicine, University of Southern Denmark, Odense, Denmark

## Abstract

Induced pluripotent stem cells (iPSCs) are becoming an important source of pre-clinical models for research focusing on neurodegeneration. They offer the possibility for better understanding of common and divergent pathogenic mechanisms of brain diseases. Moreover, iPSCs provide a unique opportunity to develop personalized therapeutic strategies, as well as explore early pathogenic mechanisms, since they rely on the use of patients’ own cells that are otherwise accessible only post-mortem, when neuronal death-related cellular pathways and processes are advanced and adaptive. Neurodegenerative diseases are in majority of unknown cause, but mutations in specific genes can lead to familial forms of these diseases. For example, mutations in the superoxide dismutase 1 gene lead to the motor neuron disease amyotrophic lateral sclerosis (ALS), while mutations in the *SNCA* gene encoding for alpha-synuclein protein lead to familial Parkinson’s disease (PD). The generations of libraries of familial human ALS iPSC lines have been described, and the iPSCs rapidly became useful models for studying cell autonomous and non-cell autonomous mechanisms of the disease. Here we report the generation of a comprehensive library of iPSC lines of familial PD and an associated synucleinopathy, multiple system atrophy (MSA). In addition, we provide examples of relevant neural cell types these iPSC can be differentiated into, and which could be used to further explore early disease mechanisms. These human cellular models will be a valuable resource for identifying common and divergent mechanisms leading to neurodegeneration in PD and MSA.

## Introduction

The group of synucleinopathies consists mainly of three neurodegenerative diseases: dementia with Lewy bodies, Parkinson’s disease (PD) and multiple system atrophy (MSA), the last being divided into Parkinsonian type MSA with degeneration of nigro-striatal dopamine neurons, and cerebellar type MSA with ataxic symptoms. The diseases share the same hallmark: intracellular aggregates composed in majority of a protein called alpha-synuclein (aSYN). The *SNCA* gene encodes for aSYN. When *SNCA* is mutated or multiplied, it leads to an early onset familial PD.^1,2^ Interestingly, while aSYN aggregates are found in neurons in dementia with Lewy bodies and PD, they are located in oligodendrocytes in MSA.^3^

Naturally, *SNCA* is expressed in neurons. A recent study from our laboratory showed that *SNCA* is also expressed at early stage of oligodendrocyte development.^4^ The role of aSYN in oligodendrocytes largely remains to be clarified. It is thought that under pathological condition, aSYN protein can aggregate in neurons and over time form Lewy bodies.^5^ Moreover, it was shown in experimental models that aSYN aggregates are toxic to neurons,^6,7^ and that a neuroblastoma cell line over-expressing human recombinant *SNCA* releases factors including aSYN, leading to glial reactivity,^8^ suggesting aSYN aggregates may cause neuronal injury. However, recent work evidenced that neuronal dysfunction and protein aggregation may be two independent events.^9^ Thus, although the progression of Lewy pathology throughout the brain may be due to a prion-like mechanism of cell-to-cell transfer of aSYN,^10^ it remains unclear what the initial molecular cascades leading to neuronal dysfunction are, and how they differ depending on the genetic background of the patients.

The reprogramming of human somatic cells using “stemness” transcription factors into induced pluripotent stem cells (iPSC)^11^ has revolutionized our way to approach scientific problems related to human diseases. Importantly, this discovery offers unlimited access to patient cells, which can subsequently be differentiated into relevant cell types to study early pathogenic mechanisms of neurodegeneration.^12–17^ Such iPSC-based research strategies could lead to the discovery of new therapeutic targets, biomarkers, and the development of humanized high-throughput models for drug discovery and environmental chemical safety assessment.^12–17^ Thus far, several studies utilizing iPSC-based models reported neuronal dysfunction reminiscent to mutations in PD-linked genes *LRRK2* (leucine-rich repeat kinase 2), *PINK* (PTEN-induced putative kinase 1), and *PARK2* (encodes PARKIN),^18–21^ or in the acid beta-glucocerebrosidase gene (*GBA1*), which encodes a lysosomal enzyme that is deficient in Gaucher’s disease, and which renders a risk of developing PD.^22,23^ Therefore, iPSCs appear to be robust models for understanding early pathogenic events occurring in familial forms of PD. Importantly, iPSCs may provide an ideal platform for studying diseases where no genetic cause has thus far been identified, such as MSA and idiopathic PD.^12–17^

**Figure 1:**
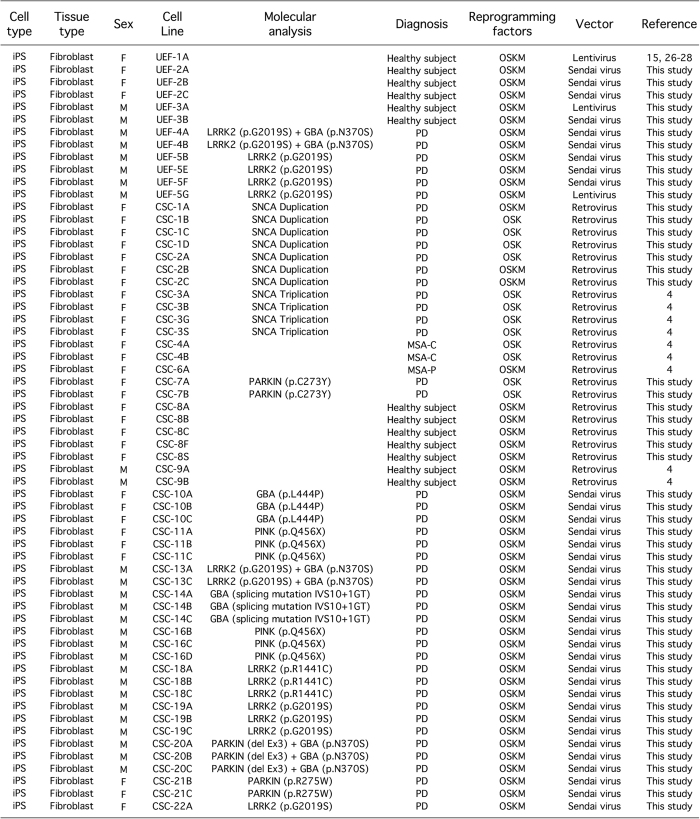
Summary of human induced pluripotent stem cells generated. A total of 61 induced pluripotent stem cells (iPSC) lines are described in this study. These were reprogrammed by retroviral, lentiviral, or Sendai virus transduction of ‘Yamanaka’ factors. All iPSC lines were generated from human dermal fibroblasts. Somatic cells were sampled from individuals diagnosed with Parkinson’s disease or multiple system atrophy, and healthy controls. The iPSC lines UEF-1A, CSC-3A, B, G and S, -4A and B, -6A and -9A, and B were previously characterized in refs 4, 15, 26–28.

The generations of libraries of familial human ALS iPSC lines have been described;^24,25^ to date, none have been described for PD. Here, we report the generation of iPSCs carrying various mutations in PD-associated genes, as well as iPSCs generated from patients diagnosed with MSA and healthy controls (Figure 1). The lines have been generated and characterized in two laboratories located in the Nordic countries Finland and Sweden, allowing regrouping research efforts under the umbrella of several interconnected research centers, which focus their research on neurodegeneration. We present an exhaustive list of iPSC lines, and describe the tests we employed to validate their pluripotency and final selection. Moreover, we provide examples of relevant neural cell types, e.g., midbrain neural floor-plate progenitors, dopamine neurons, astrocytes, and oligodendrocytes that the iPSC lines can be differentiated into. Altogether, these human cellular models provide a unique resource to study PD and MSA.

## Results

### IPSC lines generated in the Stem Cell Laboratory of Molecular Brain Research Group, at the University of Eastern Finland, in Finland

**Figure 2:**
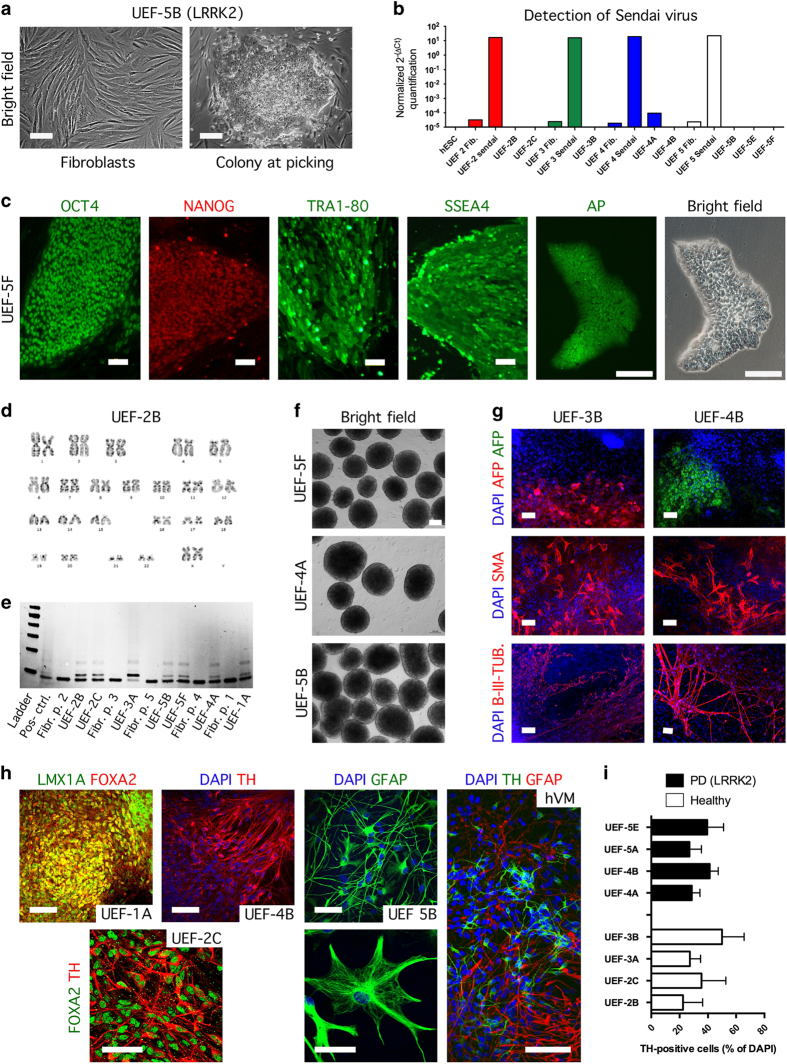
Tests of pluripotency applied to the induced pluripotent stem cells (iPSC) lines generated in the Stem Cell Laboratory of Molecular Brain Research Group, at the University of Eastern Finland (UEF), in Finland. (**a**) Representative bright field images of human fibroblasts prior to transduction and after the transduction. The putative iPSC colony shows a hES cell-like morphology. (**b**) The expression levels of Sendai virus in iPSC lines UEF-2B and C (at passage 17; 15), UEF-3B (at passage 15), UEF-4A and B (at passage 17; 15), and UEF-5B, E and F (at passage 18; 15; 16). The levels are compared to non-transduced fibroblast and to hES cells. (**c**) Representative fluorescence images of iPSC lines stained for the pluripotent markers OCT4, NANOG, TRA1–80 and SSEA4. IPSC colonies stain positive for alkaline phosphatase activity. Images are shown for iPSC line UEF-5F. Scale bars represent 100 μm. (**d**) Karyogram of iPSC line UEF-2B shows pairs of chromosomes stained using Giemsa (G-banding). (**f**) IPSC lines form embryoid bodies (EBs) grown in low-adherent plates for 2 weeks (representative image shown for iPSC line UEF-4A, 5B,F). (**g**) Differentiated EBs generate cells of the three germ layers, immunopositive for alpha-fetoprotein (AFP) (endoderm), smooth muscle antibody (SMA) (mesoderm), and beta III-tubulin (B-III-TUB) (ectoderm); nuclei are counterstained with 4’,6-diamidino-2-2phenylindole (DAPI; shown for iPS lines UEF-3B and UEF-4A). (**e**) Detection of telomerase activity using TRAPeze telomerase detection kit. (**h**) Representative images of iPSC lines differentiated towards neural, neuronal, and glial fates. IPSC lines differentiated for 20 days are positive for LMX1A and FOXA2 (midbrain neural progenitors, UEF-1A); when progenitors are kept for two additional weeks in maturation medium, they differentiate into tyrosine hydroxylase (TH)-expressing neurons (shown for iPSC line UEF-4B) that co-label with FOXA2 (shown for iPSC line UEF-2C). Undifferentiated neural progenitors cultured for 4 additional months generate glial fibrillary acidic protein (GFAP)-expressing astrocytes (shown for iPSC line UEF-5B). TH-positive and GFAP-positive cells in a human-derived ventral mesencephalic (hVM) culture served as a reference. Nuclei are counterstained with DAPI. Scale bars represent 100, 50 and 20 μm. (**i**) Differentiated iPS cells towards tyrosine hydroxylase positive (TH+) cells at day 35. Data are expressed as mean % of TH+ cells compared to DAPI±SD.

**Figure 3:**
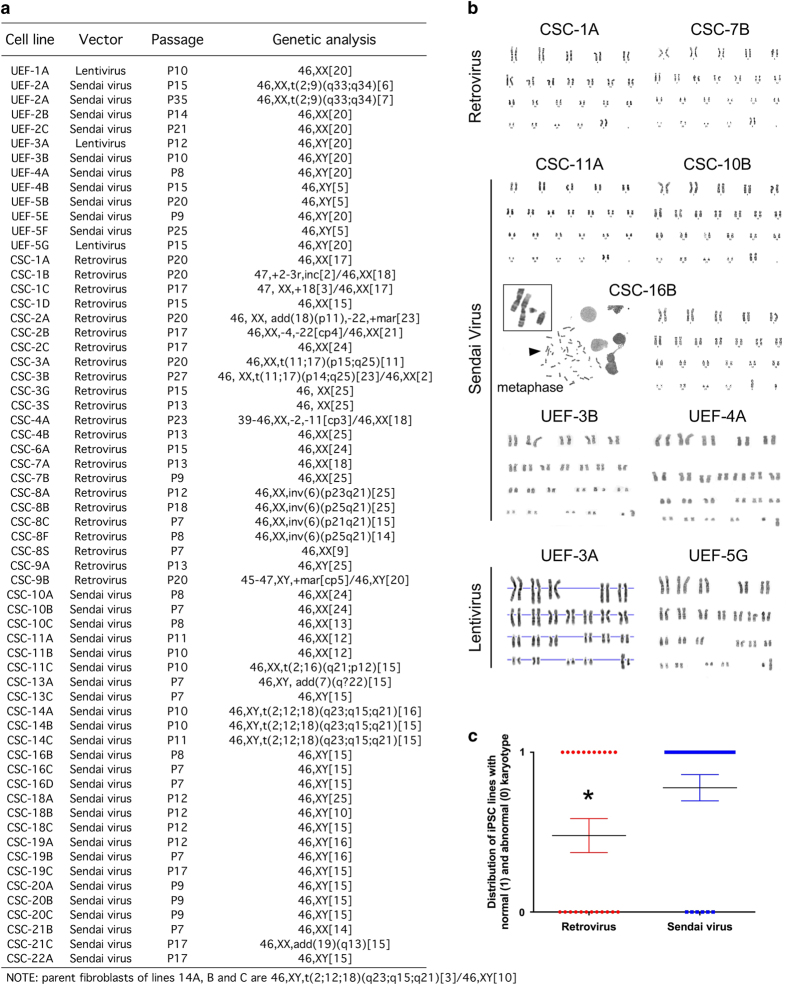
Cytogenic analysis of the induced pluripotent stem cells (iPSC) lines. (**a**) List of the iPSCs lines and corresponding karyotypes based on G-banding analysis. Note: parent fibroblasts used to generate the lines CSC-14A, CSC-14B and CSC-14C, are a mosaic of abnormal (46,XY,t(2;12;18)(q23;q15;q21) and normal (46,XY) cells. (**b**) Karyograms of iPSC lines reprogramed using retrovirus (CSC-1A and CSC-7B), Sendai virus (CSC-11A, CSC-10B, CSC-16B, UEF-3B and UEF-4A), and lentivirus (UEF-3A and UEF-5G) transduction. Representative image of metaphase (shown for CSC-16B) used for generating karyograms. (**c**) Higher numbers of iPSC lines with abnormal karyotyping are generated when parent fibroblasts are reprogrammed using retroviral transduction. Unpaired *t*-test revealed significance between the two groups compared (*P<0.05); lentivirus group was not included in the analysis because *n*=3 only. Note: analysis performed for Cell and Stem Cell (CSC) laboratory iPSC lines only; iPSC lines CSC-14A, -14B and -14C carrying the same chromosomal abnormality in all clones tested were excluded from the distribution analysis, as they reflected a constitutional chromosomal change present in the parent fibroblasts.

We present the characterization of iPSC lines generated by two reprogramming methods using either lentivirus or Sendai virus. We used the following nomenclature in description of University of Eastern Finland (UEF) lines: number=patient and letter=clone; e.g., UEF-2A→line generated at the UEF lab, patient 2, clone A. The skin biopsies were collected from individuals with confirmed diagnosis and mutation, and the fibroblast populations were transduced with viruses carrying genes encoding mouse *Oct4, Klf4, SOX2*, and *cMyc* (for UEF-1A line) or human *OCT3/4, KLF4, SOX-2*, and *c-MYC* (for UEF-3A and UEF-5G lines). The lines UEF-1A (i.e., UEFhfiPS1.4 in our previous reports^15,26–28^), UEF-3A and UEF-5G were generated with a polycistronic lentivirus carrying all the reprogramming factors in the same viral vector (STEMCCA) while the lines UEF-2A, B and C, -3B, -4A and B, and -5B, E, F and G, were transduced with four separate Sendai viruses, encoding the same pluripotency genes (Figure 1). Following the transduction with the four factors, we observed early morphological changes indicative of reprogramming (Figure 2a). An average of 3–6 embryonic stem cell (ESC)-like colonies were manually picked and expanded clonally. These clonal lines were cultured until about passage 10 prior to testing their pluripotency to ensure full maturation of hiPSCs.^29,30^ At that time point, we detected by using quantitative real-time PCR pluripotency-promoting endogenous gene expression, including *OCT3/4, SOX2, NANOG, KLF4, cMYC,* and *LIN28* in all our iPSC lines (Supplementary Figure S1), and confirmed the absence of the virally delivered transgenes (Figure 2b and Supplementary Figure S1). All iPSC lines expressed several human ESC-associated antigens NANOG, SSEA4, TRA1–81, and OCT4 (Figure 2c), and the alkaline phosphatase staining was positive (Figure 2c). Chromosomal analysis from all iPS cell lines showed the normal karyotypes 46,XX or 46,XY (Figures 2d and 3) except of UEF-2A line where translocation in chromosome 2 and 9 was detected (Figure 3). In addition, the iPSC lines showed high telomerase activity when compared with their parent fibroblasts (Figure 2e).

All lines formed embryoid bodies when plated in suspension dishes (Figure 2f). Immunocytochemical analyses of embryoid bodies were performed after 10–14 days of culture and showed that each line had spontaneously differentiated into cell types representative of the three embryonic germ layers, including alpha-fetoprotein (AFP)-positive cells (endoderm), beta III-tubulin (B-III-TUB)-positive cells (ectoderm), and smooth muscle antibody (SMA)-positive cells (mesoderm; Figure 2g).

We adapted previously published protocols^31,32^ to generate iPSC-derived mesencephalic dopaminergic neurons. The differentiation of iPSCs was started on human feeder layer. On day 14, small pieces of colonies were plated in an adherent culture. On day 20, the neuroepithelium differentiated into cells resembling neural cells. The neuroprogenitors were positive for LMX1A and FOXA2, confirming they acquired a midbrain floor-plate identity (Figure 2h). After 2 weeks of maturation, ~40% of all cells were strongly immunoreactive for tyrosine hydroxylase (Figure 2i), and resembled tyrosine hydroxylase-positive neurons found in cultures derived from 5-week-old human embryonic ventral mesencephalic tissue (Figure 2h). Differentiation of astroglial progenitors and astrocytes from the iPSCs lines was adopted from a previously published protocol.^33^ Using this method we were able to generate a homogenous population of astrocytes within 4 months (Figure 2h).

### IPSC lines generated in the Cell and Stem Cell Laboratory for CNS Disease Modeling, at the University of Lund, in Sweden

**Figure 4:**
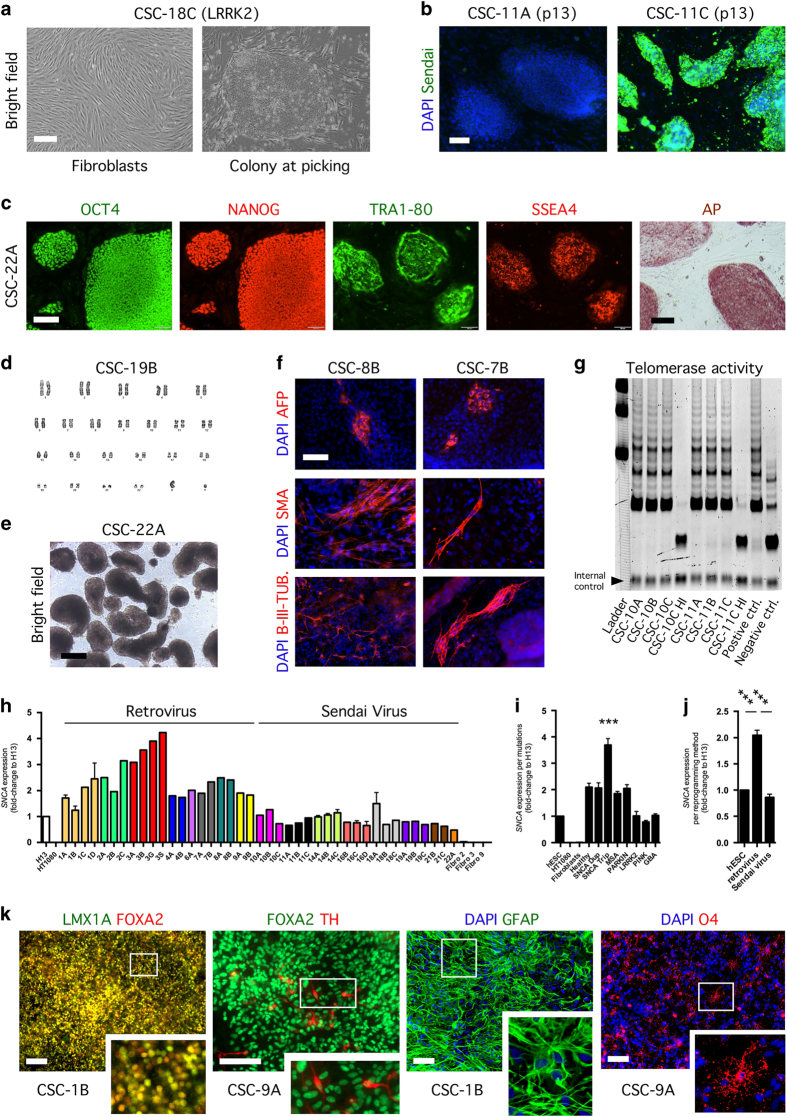
Tests of pluripotency applied to the induced pluripotent stem cells (iPSC) lines generated at the Cell and Stem Cell (CSC) Laboratory for CNS disease modeling, at the University of Lund, in Sweden. (**a**) Prior to transduction, human fibroblasts display a bipolar elongated shape. Upon transduction, putative iPSC colonies show a human pluripotent stem cell-like morphology. Scale bars represent 100 μm. (**b**) Loss and maintenance of Sendai virus in iPSC lines CSC-11A (at passage 13) and CSC-11C (at passage 13), respectively. Nuclei are counterstained with DAPI. Scale bar represents 100 μm. (**c**) Representative images of iPSC lines stained for the pluripotent markers OCT4, NANOG, TRA1–80 and SSEA4. IPSC colonies stain positive for alkaline phosphatase activity. Images are shown for iPSC line CSC-22A. Scale bars represent 100 μm. *n*=2–3 independent experiments. (**d**) Karyogram of iPSC line CSC-7B shows pairs of chromosomes stained using Giemsa (G-banding). (**e**) IPSC lines form embryoid bodies (EBs) when grown in low-adherent surfaces for 2 weeks, in WiCell Medium supplemented with FGF2 (representative image shown for iPSC line CSC-22A). Scale bar represents 100 μm. *n*=2–3 independent experiments. (**f**) Differentiated EBs generate cells of the three germ layers, immunopositive for alpha-fetoprotein (endoderm), smooth muscle antibody (mesoderm), and beta III-tubulin (ectoderm); nuclei are counterstained with DAPI. Scale bar represents 100 μm. *n*=2–3 independent experiments. (**g**) Detection of telomerase activity by the TRAP assay. (**h**) Upregulation of *SNCA* expression in clonal iPSC cell lines CSC-3A, -3B, 3G and 3S, revealed by quantitative real-time PCR. Values are normalized to house keeping gene GAPDH and calibrated to marker expression in human embryonic stem cell (hESC) line H13. Mean±s.e.m. shown for *n*=2 independent experiments. (**i**) Upregulation of *SNCA* expression in SNCA triplication iPSC lines revealed by quantitative real-time PCR. One-way analysis of variance (ANOVA; *P*>0.0001, F_(10;49)_=30.03) followed by Dunnett’s multiple comparisons tests shows SNCA triplication lines have a significantly (***P<0.0001) higher *SNCA* expression over non-SNCA triplication lines. Mean±s.e.m. (**j**) Higher expression of SNCA in lines generated with retrovirus transduction over those generated with Sendai virus transduction. One-way ANOVA (*P*>0.0001, F_(2;29)_=58.36) followed by Tukey’s multiple comparisons test shows lines reprogramed using retrovirus transduction have a significant (^***^P<0.0001) higher basal level of *SNCA* expression, when compared with SNCA expression levels of hESC line H13 and iPSC lines generated using Sendai virus transduction. Mean±s.e.m. (**k**) Representative images of iPSC lines differentiated towards neural, neuronal, and glial fates. IPSC lines differentiated for 12 days become midbrain neural progenitors co-expressing LMX1A and FOXA2 (shown for iPSC line CSC-1B); when progenitors are kept for 4 additional weeks in culture, they differentiate into FOXA2/tyrosine hydroxylase-expressing neurons (shown for iPSC line CSC-3G; aged 30 days *in vitro*). Culturing of the remaining undifferentiated neural progenitors for 5 additional weeks generates glial fibrillary acidic protein-expressing astrocytes. O4-positive oligodendrocytes can be generated from iPSC using medium devoid of retinoic acid, as we previously published.^4^ Nuclei are counterstained with DAPI. Scale bars represent 100 μm. Images are representative of *n*=1–2 independent experiments.

We present the characterization of iPSC lines generated using two distinct methods of reprogramming. All iPSC lines were generated from skin fibroblasts of individuals with confirmed diagnosis, obtained from open access resources. We adapted a protocol similar to that recently described,^34^ allowing us to reprogram up to 12 patient fibroblast samples at once, minimizing associated costs. The nomenclature used is similar to that implemented by UEF: number=patient and letter=clone; e.g., CSC-2A→line generated at the Cell and Stem Cell (CSC) lab, patient 2, clone A. The lines CSC-1, -2, -3, -4, -6, -7, 8-, and -9 were generated by retroviral transduction of 3 (OCT3/4, SOX2, KLF4) or 4 (the 3 plus c-MYC) factors; while the lines CSC-10, -11, -13, -14, -16, -18, -19, -20, -21, and -22 were generated by Sendai virus transduction of the four factors (Figure 1). Putative iPSC colonies were picked only if presenting a well-defined pluripotent stem cell-like morphology (Figure 4a). Unless fibroblasts displayed low reprogramming efficiency, an average of 24 colonies was picked per patient reprogrammed fibroblasts. These were subsequently expanded onto irradiated mouse embryonic fibroblasts (using a 24-well plate format) for up to 4 passages, prior to being cryopreserved.

We commonly thaw 3 putative iPSC clones for each patient lines generated, grow all clonal cell lines in 6-well plates for up to 10 (some times more) passages (with one simultaneous passage per week), and perform within the latest passages, in addition to finger print analysis, a battery of tests allowing us to determine the efficiency of reprogramming from putative to well-established/mature iPSC lines. The tests comprise: (1) expression of nuclear and cell surface markers reminiscent to pluripotent stem cell stage (OCT4, NANOG, TRA1–80 and SSEA4), (2) alkaline phosphatase activity, (3) telomerase activity, (4) embryoid body formation, (5) loss of viral agents or downregulation of viral transgenes, and (6) differentiation towards the three germ layers. The lines can further be tested for their ability to generate midbrain neural cells types: LMX1A/FOXA2 co-expressing neural floor-plate progenitors, FOXA2/tyrosine hydroxylase-expressing neurons, glial fibrillary acidic protein-expressing astrocytes, or O4-positive oligodendrocytes, which can be used for studies of development and disease phenotypes. All these tests are performed *in vitro*. In addition, the lines are tested for their karyotype stability, and the tests are performed including a human ESC line (HuES3 and/or HuES13) as positive control. In some assays, e.g., determination of telomerase activity, both embryonic stem cells and parent fibroblasts can be used as positive or negative controls, as we previously reported,^4^ as well as heat-inactivated samples.

**Figure 5:**
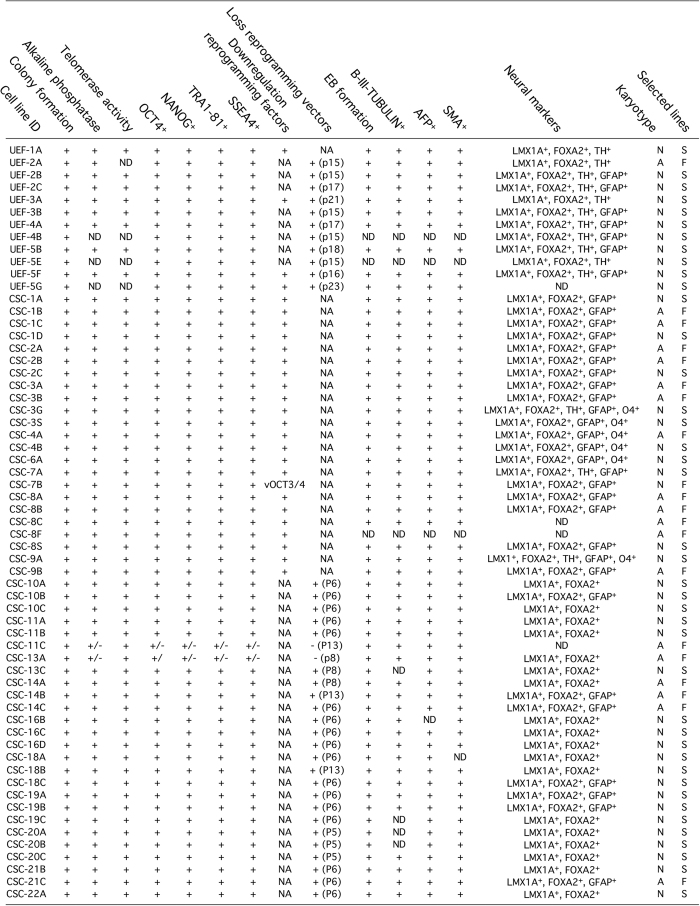
Summary of human induced pluripotent stem cells generated. Summary of the assays employed for characterizing the iPSC lines. The iPSC lines UEF-1A, CSC-3A, B, G and S, -4A and B, -6A and -9A and B were previously characterized in refs.^4,15,26–28^ A, abnormal; F, failed; N, normal; NA, not applicable, ND, not determined; (Pn), passage when karyotyping was performed; S, selected; +, successfully passed; +/−, presence of marker of interest.

Sendai virus transduction is advantageous in many ways, as it leads to high efficiency reprogramming when compared with retroviral delivery method of reprogramming, and it is non-integrative.^35^ Interestingly, two clonal cell lines (CSC-11C at passage 13 and CSC-13A at passage 8) showed resistance to Sendai virus elimination over passages (Figure 4b and Figure 5). Consequently, these failed reprogrammed clones only partially passed most of the tests (Figure 5). Importantly, all other lines, including those reprogrammed using retroviral transduction, showed immunoreactivity for the pluripotency markers OCT4, NANOG, TRA1–80, and SSEA4. Almost all lines had completely downregulated viral transgene expression, with the exception of line CSC-7B, which had a strong expression of viral *OCT4* (Figure 4c, Supplementary Figure S2, and Figure 5); moreover, the lines displayed a robust alkaline phosphatase activity (Figure 4c), which we observed weaker for CSC-11C and CSC-13A. Although we did not perform rigorous side-by-side comparisons of the reprogramming methods, we observed that reprogramming by means of Sendai virus transduction compared to retrovirus transduction led to less karyotyping abnormalities (Figure 4d and Figure 3), as recently described.^36^ Nevertheless, the clonal cell lines could generate embryoid bodies when cultured on non-adherent surfaces, in Wicell medium supplemented with basic fibroblast growth factor (Figure 4e), and subsequently differentiate into all three embryonic germ layers when exposed to fetal bovine serum, as detected by the presence of AFP-positive, SMA-positive, and B-III-TUB-positive cells, after 2 weeks *in vitro* (Figure 4f). Finally, we confirmed high telomerase activity for the iPSCs (Figure 4g), as well as higher expression of *SNCA* gene for the clones CSC-3A, -3B, -3G, and -3S (*SNCA* triplication), regardless whether they were generated using three or four factors and if they carried karyotype abnormalities (Figure 4h and Figure 3). Interestingly, the iPSC lines reprogrammed by retroviral transduction displayed higher basal level of expression of *SNCA* gene compared with hESC control and iPSC lines generated by Sendai virus transduction; this observation required the exclusion from the analysis of the lines harboring duplication and triplication of *SNCA* gene (Figure 4j), and may underlie a possible leakiness in expression of the integrated transgenes (Supplementary Figure S2).

We adapted our previous published protocols^4,37^ to generate iPSC-derived neural progenies. Thus, we could generate midbrain neural floor-plate LMX1A/FOXA2-co-expressing progenitors, which after subsequent differentiation gave rise to FOXA2/tyrosine hydroxylase-expressing neurons and glial fibrillary acidic protein-expressing astrocytes (Figure 4k). IPSCs could also be differentiated into O4-positive oligodendrocytes (Figure 4k).

## Discussion

It was only less than a decade ago that successful reprogramming of somatic cells into ESC-like cells was reported. Two initial studies described that treating permeabilized human 293T cells with carcinoma nuclear cell extract, and overexpression of four pluripotent genes in dividing mouse fibroblasts, could reprogram somatic cells back to an ESC-like pluripotent stage.^38,39^ The first method was adapted from previous report of the same group, showing that human 293T cells expressed T-cell functions when reprogrammed using primary human T-cell extract.^40^ The second method, employing overexpression of rodent pluripotent genes *Sox2*, *Oct4*, *Klf4,* and *c-Myc*, allowed large-scale generation of the so-called iPSCs, and is now routinely used for the reprogramming of patient somatic cells for ‘modeling diseases in the dish’.

Various techniques exist to generate iPSC; they are commonly subdivided into genome-integrating and genome non-integrating approaches. These technologies rely on the use of different vectors^35^ and they have been extensively studied and improved during the last years.^41,42^ Moreover, much effort to identify chemical compounds that could substitute the reprogramming factors and the use of vectors is ongoing.^43^ Interestingly, when we compared the expression of *SNCA* in iPSC lines generated using two different approaches, we found higher level of *SNCA* expression in iPSC lines generated using a genome-integrating approach, when compared with iPSC lines generated by Sendai virus transduction; this analysis excluded the lines carrying multiplications of the gene of interest. Thus, it will be important in subsequent studies to verify that variability is not added by the reprogramming method employed to generate the iPSCs, to that already existing amongst the different clonal cell lines generated from a single patient biopsy sample. This may prevent the identification of subtle phenotypes if too few lines are analyzed, but could be circumvented by the use of isogenic lines, which appears to be the most appropriate control since gene mutation correction allows reversing disease phenotypes.^44,45^

We also demonstrated differentiation of our iPSC library lines not only into midbrain dopaminergic neurons but also to relatively pure astrocyte and enriched oligodendrocyte cultures. Considering that non-neuronal cells, not limited to oligodendrocytes in MSA, contribute to the PD pathology, our iPSC library will allow for disease mechanism analyses and drug screens practically with all cell types involved in development of PD. Three-dimensional cell culture models^46–48^ and transplantation of iPSC-derived neural cells into the rodent brain^15,49–54^ are still other promising strategies to be pursued using PD-iPSC library lines for revealing the contribution of genetic factors to this disease.

IPSC-based models provide an unprecedented opportunity to study rare diseases of the brain where genetic causes have not been identified yet, and offer the possibility to develop new diagnostic tools for early diagnosis, as well as for the prospective stratification and recruitment of patients for future clinical trials. Importantly, iPSCs provide a source of terminally differentiated cells that can be used to develop completely humanized assays for small to high throughput or high-content screens of neuroprotective compounds^13,17^ and validation of drug candidates previously identified using non-relevant cell models.^55^ Taking example of existing Lab-on-chip platforms, iPSC research could rapidly develop towards a Lab-on-iPSC platform or Pharmaco-iPSCellomics by person-specific iPSCs, as previously reported.^12^ Because of their pluripotency, iPSCs retain the ability to differentiate into multiple cell types of the three germ layers. Consequently, compounds identified as potential neuroprotective drug candidates could be tested in patient iPSC-derived hepatocytes and cardiomyocytes for possible toxicity, providing strong pre-clinical data and a higher degree of confidence prior to starting clinical trial, as well as a greater level of safety for the patients. The implementation of such ambitious projects will require the use of high-throughput platforms and stringent standard operative procedures not always available in academic institutions. It is likely that these projects will have to be conducted in large consortia, and in collaboration with industrial partners,^56,57^ as their cost would be enormous, especially if combinations of several drug candidates are tested.

In this study, we reprogrammed human fibroblasts from healthy subjects and patients diagnosed with PD and MSA. We applied a battery of quality control tests, which allowed us to select 41 iPSC lines for follow up studies, out of the 61 iPSC lines we initially characterized. We excluded lines that displayed abnormal karyotyping (*n*=19), strong expression of reprogramming factor due to transgene insertion (line CSC-7B), and persistence of Sendai virus (lines CSC-11C and CSC-13A). In summary, we obtained 8 “good” control iPSC lines and 33 “good” disease iPSC lines (Figure 5).

We are continuously generating new iPSC lines using somatic cells from individuals diagnosed with synucleinopathies, including idiopathic PD cases. We have also extended our capabilities to other neurodegenerative diseases such as Alzheimer’s disease. These models will allow us to identify mechanisms that are truly disease specific to those commonly engaged in an adaptive response to neurodegeneration. Such approach, together with ongoing efforts in the field, should allow gaining insights into neurodegenerative processes, and help identify proper targets for therapeutic interventions and allow “GWA”-like iPSC studies for the discovery of new biomarkers and patient stratification.

## Materials and methods

### Use of animals and human samples

All procedures were conducted in accordance with national and European Union directives. The generation of human iPSC lines using viral-mediated gene delivery was approved by the ethical committee for the use of laboratory animals at Lund University and the ethical committee on Research Ethics of Northern Savo Hospital district, Finland, as well as the Swedish Work Environment Authority (Arbetsmiljö verket).

### Statistical analyses

All quantitative data was analyzed using Prism 6.0 (GraphPad, La Jolla, CA, USA). Sample groups were subjected to one-way analysis of variance and unpaired t-test. A *P*-value of <0.05 was considered significant.

### Experimental procedures

Details methods are described in Supplementary Methods, and published in refs 4,15,26 and 37.
