# Supplementary material for: Creation of a library of induced pluripotent stem cells from Parkinsonian patients
Source: NPJ Parkinsons Dis. 2016 Jun 2;2:16009–. doi: 10.1038/npjparkd.2016.9 (PMC5516589; doi:10.1038/npjparkd.2016.9)
Supplement: Supplementary Information [file npjparkd20169-s1.doc]

**Material and Methods**

***IPSC lines generated in the Stem Cell laboratory of Molecular Brain Research Group, at the UEF, in Finland.***

*The human subjects*

The human subjects participating in this study were recruited from Kuopio University Hospital (Kuopio, Finland; Approved by the committee on Research Ethics of Northern Savo Hospital district, license no. 42//2010) and patients of Neurology Center cooperating with Vavilov Institute of General Genetics, Russian Academy of Science, Moscow, Russia. Written informed consent was obtained from the study participants. In total 5 individual subjects participated in the study. Two of these had been diagnosed with familial PD and they carried G2019S mutation in LRRK2 gene (named as UEF-4, -5 hereafter) the most common PD-related mutation. In addition, UEF-4 patient carried another mutation, which was the substitution N370S in glucocerebrosidase (GBA) gene. The mutations in LRRK2-G2019S and GBA-N370S were confirmed by direct DNA sequencing using the genetic analyzer (ABI Prism 3130, Applied Biosystems) and by multiplex polymerase chain reaction (PCR) in reverse transcriptase using nucleic acid analyzer (ANC-32, Synthol). Another 3 healthy individuals, with no family history of PD, were chosen as controls (UEF-1, -2, -3).

*Derivation of induced pluripotent stem cells*

Skin biopsy-derived fibroblasts were reprogrammed either with STEMCCA polycistronic lentivirus (Millipore) or with CytoTune® iPS Sendai Reprogramming kit with slight modifications (Thermo Fisher Scientific). Briefly, fibroblasts (1x10^5^), originating from healthy adult volunteers or the PD patients were seeded in 35-mm-well in fibroblast culture medium (FM) containing Iscove’s DMEM, 1% non-essential amino acids, 100 IU/ml penicillin and 100 μg/ml streptomycin (all from Thermo Fisher Scientific) and 20% heat-inactivated foetal bovine serum (FBS; Biowest, MO, US) on day 0. Next day, the fresh fibroblast culture medium was changed and STEMCCA polycistronic lentivirus containing m*Oct-4, Klf-4, Sox-2, and c-Myc* (OKSM) or CytoTune® iPS Sendai Reprogramming kit (Thermo Fisher Scientific), containing four separate vectors carrying genes h*OCT-3/4, hKLF-4, hSOX-2 and hc-MYC* (multiplicity of infection value being 4 for each), was used for induction of pluripotency. One day later, fresh FM was changed and 0.5 mM sodium butyrate (Sigma-Aldrich) was added to the medium to enhance the reprogramming ^1^. Medium was supplemented with sodium butyrate up to 10 days and changed every other day. One week after transduction, 0.75x10^5^ cells were seeded on the top of mitotically inactivated (10 µg/ml mitomycin-C for 2.5 h in 37°C) human foreskin fibroblast (CRL-2429, ATCC, Manassas, VA) feeder cells growing in 10 cm petri dish ^2^. On the following day, fibroblast culture medium was replaced with the human pluripotent stem cell (hPSC) culture medium ^3^, including basic fibroblast growth factor (10 ng/ml). Thereafter, medium was changed and growth of colonies was observed daily. First colonies started to appear a week later, and they were cloned by picking up individual colonies, and re-seeding them separately into a 35 mm plate containing fresh feeder cells. From each study subject´s transduced cell colonies, 3-6 clones were collected. Sub-culturing was done once a week and medium was changed daily.

*Characterization of hiPSC pluripotency*

The pluripotency of created hiPSC lines was assessed as earlier in our studies (Qu et al., 2013) with slight modifications. Briefly, the cell and colony shape and growth were followed. TaqMan gene expression assays (Thermo Fisher Scientific) containing 6-carboxyfluorescein (FAM) or VIC dye were used to measure the RNA expression of NANOG (Hs02387400_g1), LIN 28 (Hs00702808_s1), OCT-4 (Hs00742896_s1), SOX-2 (Hs01053049_s1), c-MYC (Hs00153408_m1) and KLF-4 (Hs00358836_m1) normalized to β-ACTIN (Thermo Fisher Scientific) and compared to non-transduced initial fibroblasts. For PCR or RT-PCR, PCR amplification was performed using Taq polymerase (Thermo Fisher Scientific) according to the manufacturer protocol in a thermocycler (Eppendorf). PCR products were analyzed by electrophoresis in 1% agarose gel. The list of primers used in this study is summarized in the table below.

| Primer | Direction | Sequence (5' to 3') | Tm (°C) | Source |
| --- | --- | --- | --- | --- |
| endogenous  mc-Myc | Forward (F)  Reverse (R) | TCAAGCAGACGAGCACAAGC TACAGTCCCAAAGCCCCAGC | 60 | This report |
| endogenous  mKlf-4 | F  R | GGCGAGAAACCTTACCACTGT TACTGAACTCTCTCTCCTGGCA | 60 | This report |
| endogenous  mSox-2 | F  R | TCTGTGGTCAAGTCCGAGGC CTTCTCCAGTTCGCAGTCCAG | 60 | This report |
| endogenous  mOct-4 | F  R | CCAACGAGAAGAGTATGA CAAAATGATGAGTGACAG | 60 | This report |
| endogenous  mβactin | F  R | ATGCTCCCCGGGCTGTAT  CATAGGAGTCCTTCTGAC | 60 | This report |
| endogenous  hβACTIN | F  R | CCTGGCACCCAGCACAAT  GGGCCGGACTCGTCATAC | 60 | This report |
| endogenous  hOCT-4 | F  R | CGACCATCTGCCGCTTTGAG  CCCCCTGTCCCCCATTCCTA | 69 | This report |
| endogenous  hSOX-2 | F  R | TCCTGATTCCAGTTTGCCTC  GCTTAGCCTCGTCGATGAAC | 69 | This report |
| endogenous  hNANOG | F  R | CAGCCCTGATTCTTCCACCAGTCCC  TGGAAGGTTCCCAGTCGGGTTCACC | 69 | This report |
| PARK8 | F  R | GTGGACATTTATATTTAAGG  GATGCTTGCATTTTTTCAC | 60 | This report |
| transgenic  hOCT-4 | F  R | CGACCATCTGCCGCTTTGAG  CGAAGTTATTAGGTCCCTCG | 66 | This report |
| transgenic  hSOX-2 | F  R | TCCTGATTCCAGTTTGCCTC  CGAAGTTATTAGGTCCCTCG | 66 | This report |
| transgenic  hKLF-4 | F  R | GCGCTGCTCCCATCTTTCT  CGAAGTTATTAGGTCCCTCG | 66 | This report |
| transgenic  hc-MYC | F  R | AGTAATTCCAGCGAGAGGCA  CGAAGTTATTAGGTCCCTCG | 55 | This report |

The expression level of Sendai virus and thus its existence in the created hiPSC-lines was checked at approximately passage 15. Transcription factors Oct-4 (MAB4401, Millipore, Billerica, MA) and Nanog (AF1997, R&D Systems, Minneapolis, MN), cell surface markers SSEA-4 (MAB4304, Millipore) and Tra-1-81 (MAB4381, Millipore) ^3^ and alkaline phosphatase activity (alkaline phosphatase live stain, Thermo Fisher) were determined at protein level by immunostaining. Karyotyping was performed using Giemsa (G-banding) staining, after arresting the cells in the metaphase by 200 ng/ml N-desacetyl-N-methylocolchicine. The property to form embryoid bodies (EBs) was checked by growing the hiPSCs in low-adherent plates for 2 weeks after which the EBs were plated down for 2 weeks and the expression of proteins originating from the 3 germ layers were checked, i.e. alpha fetoprotein (AFP) for endoderm, smooth muscle actin (SMA) for mesoderm, and beta-III-tubulin (B-III-TUB) for ectoderm. Telomerase activity was determined by the telomeric repeat amplification method using TRAPeze telomerase detection kit (Merck Millipore).

*Dopaminergic neurons and astrocytes*

Ventral midbrain dopaminergic neurons were differentiated as described earlier (Kriks et al., 2011; Xi et al., 2012) with slight modifications. Human iPSC colonies were maintained on human foreskin fibroblasts in human embryonic stem hESC medium (Ström et al., 2007; Puttonen et al., 2013) that was gradually shifted to neural induction medium (NIM; Puttonen et al., 2012) starting on day 5 of differentiation. For the first 5 days, SB 431542 (20 µM) and LDN 193189 (100 nM) (both from Miltenyi Biotec) were added to the medium. To pattern the cells to the midbrain progenitors, cells were exposed to recombinant human fibroblast growth factor 8b (FGF8b, 100 ng/ml; Peprotech), recombinant human sonic hedgehog (SHH, 100 ng/ml; Peprotech) and Purmorphamine (2 µM; Santa Cruz Biotechnology) until day 12. On day 3, CHIR99021 (3 µM; Axon Medchem), a potent inhibitor of glycogen synthase kinase 3β (GSK3β) and activator of wnt signaling ^4,5^ was added to the culture. At day 14, the small pieces of colonies were cut and plated onto Matrigel (10 µl/ml; BD Biosciences)/fibronectin (1 mg/ml; Sigma-Aldrich) in differentiation medium (NIM) supplemented with recombinant human brain derived neurotrophic factor (BDNF, 20 ng/ml; Peprotech), recombinant human glial-derived neurotrophic factor (GDNF, 20 ng/ml; Peprotech), L-ascorbic acid (AA, 200 µM; Sigma-Aldrich), db-cAMP (0.5 mM; Sigma-Aldrich), recombinant human transforming growth factor β3 (TGFβ3, 1 ng/ml; Peprotech) and **N-[N-(3,5-Difluorophenacetyl)-L-alanyl]-S-phenylglycine t-butyl ester** (DAPT, 10 µM; Sigma-Aldrich) to support the maturation of progenitors to dopaminergic neurons. Medium was changed every other day and the samples from different time points were collected and analyzed as described below. Differentiation of astroglial progenitors and astrocytes from hiPSCs was adopted from previously published protocol by Krencik and coworkers ^6^. The cells were fixed with 4% PFA for 20 min, permeabilized with 0.25% Triton X-100 and blocked for 60 min with 5% normal goat serum-PBS. Subsequently, cells were incubated with antibody against LMX1A (rabbit, Millipore, 1:800), FOXA2 (mouse monoclonal, Santa Cruz; 1:50), TH (rabbit, Sigma-Aldrich, 1:500) and GFAP (rabbit, Dako, 1:800) overnight at 4˚C. For visualization of the primary antibodies, incubation with Alexa Fluor goat-anti mouse 488 or 568 and goat anti-rabbit 488 or 568 secondary antibodies (1:300) was followed by 4',6-diamidino-2-phenylindole (DAPI) staining.

***Material and methods used at the Cell and Stem Cell (CSC) Laboratory for CNS disease modeling, at the University of Lund, in Sweden***

*Culture of human fibroblasts*

Human fibroblasts were collected by punch skin biopsy from healthy individuals and individuals diagnosed with PD and MSA. Diagnosis of primary degenerative parkinsonism fulfilled current criteria for probable PD or MSA ^7,8^. The fibroblast cell lines used to generate iPSC lines CSC-21B, CSC-21C and CSC-22A were commercially obtained from the Coriell institute. The mutations (PARKIN p.C253Y, PARKIN p.R275W, LRRK2 p.G2019S, LRRK2 p.R1441C, GBA p.L444P, GBA p.N370S and PINK p.Q456X) were confirmed by direct DNA sequencing (Eurofins Genomics).

Cells were cultured and expanded in fibroblast growth medium composed of DMEM and 10% fetal bovine serum (Thermo Fisher Scientific) prior to cryo-banking. Only low passage fibroblasts (<8 passages) were used in the generation of the iPSC lines; all tested negative for mycoplasma.

*Derivation of induced pluripotent stem from human fibroblasts*

Proliferating fibroblasts were seeded at the concentration 1x10^5^ per well of a 12 well plate. The day after, cells were transduced with the virus in fibroblast growth medium supplemented with 4μg / ml of protamine sulfate (Sigma-Aldrich) if retroviruses were used.

The CSC lines were generated using two different strategies for delivery of the Yamanaka reprogramming factors. CSC lines 1 to 9 were generated by retroviral delivery, as previously described ^9,10^. Fibroblasts were transduced twice (Day 0 and day 1) with retroviruses (MOI of 10) prepared from the plasmids pMXs-hKLF4, pMXs-hOCT3/4 and pMXs-hSOX2 (Addgene). The iPSC lines CSC-1A, CSC-2B, 2C, CSC-6A, CSC-8A, CSC-8B, CSC-8C, CSC-8F, CSC-8S, CSC-9A and CSC-9B were generated using also a fourth retrovirus prepared from the pMXs-hc-MYC plasmid (Addgene). In the second approach, CSC lines 10 to 22 were generated by using Sendai viruses delivering the four Yamanaka factors. Here CytoTune™-iPS 1.0 (for lines CSC-21B, CSC-21C and CSC-22A) and 2.0 kits were used according to manufacturer’s instructions, with minor modifications such as scaling of culture format.

Following transduction media were changed daily to fresh fibroblast growth medium until day 6 when the cells were re-seeded on irradiated mouse embryonic fibroblasts (CF-1 MEF, GlobalStem) in WiCell medium composed of advanced DMEM/F12 (Thermo Fisher Scientific), 10% Knock-Out Serum Replacement (v/v, Thermo Fisher Scientific), 2 mM L-glutamine (Thermo Fisher Scientific), 1% non-essential amino acids (NEAA, v/v, Millipore), 50 μM β-mercaptoethanol (Sigma-Aldrich) and 20 ng/ml FGF2 (Thermo Fisher Scientific). Colonies were picked based on morphology 2-4 weeks following seeding of the transduced fibroblasts on MEFs. For a few of the lines additional MEFs where added to the cultures. Each clone was individually expanded and bio-banked waiting testing of pluripotency.

*Spontaneous differentiation of iPSC into the three germ layers*

Human iPSC were grown for 2 weeks as embryoid bodies (EBs) in low-attachment multiwell plates (Corning) in WiCell with 20 ng/ml FGF2. The EBs were then re-seeded in 96-well plates (Greiner Bio-One) coated with 0.1% gelatin (Millipore) in DMEM medium containing 10% fetal bovine serum, 1% penicillin and streptomycin (v/v, Thermo Fisher Scientific), and for subsequent spontaneous differentiation over two weeks. Media were changed every 2-3 days.

*Differentiation of iPSCs into neural progenies*

One day prior to the start of differentiation, human iPSC colonies were harvested, dissociated, and seeded on a Matrigel-coated (BD Bioscience) 96-well plates in WiCell medium supplemented with 20 ng/ml FGF2 and 20 μM Y-27632 (Reagents Direct). The day after (counted as day 0/D0), the media was changed to neural induction medium containing advanced DMEM/F12 and Neurobasal medium (1:1), B27 minus vitamin A (v/v, 2% D0-D2 and 1% D4-10), N-2 (v/v, 1% D0-D2 and 0.5% D4-10), 2 mM L-glutamine and 1% P/S (Thermo Fisher Scientific). To direct differentiation towards midbrain neuronal progenitor lineage, we supplemented the medium with 0.1 μM LDN (Stemgent, D0-D8), 10 μM SB (Reagents Direct, D0-D4), 200 ng/ml SHH-C (Thermo Fisher Scientific, D0-D10), 0.8 μM CHIR (Stemgent, D0-D10) and 1 μM Smoothened agonist (Millipore, D2-D10). Media were changed every other day. Cells were fixed with 4% paraformaldehyde on day 12 of differentiation for subsequent immunocytochemistry.

To generate TH-expressing neurons, floor-plate progenitors were generated as EBs, using the abovementioned protocol, then dissociated on D12 and plated at high density on plates coated with 20 μg/ml Poly-ornithinin (PO, Sigma-Aldrich) and 50 μg/ml Laminin (Thermo Fisher Scientific). Neuronal differentiation medium composed of Neurobasal, 2% B27 minus vitamin A, 2mM L-glutamine and 1% P/S was added every 2-3 days. This medium was supplemented with 20 ng/ml BDNF (R&D), 10 ng/ml GDNF (R&D), 5 ng/ml CDNF (Thermo Fisher Scientific), 200 μM Ascorbic Acid (AA), 500 μM db-cAMP (Sigma-Aldrich) to induce differentiation into TH-positive cells. The cells were matured for two weeks, and fixed on day 30 for immunocytochemistry.

To generate glial cells, EBs containing regionalized neural progenitors were expanded for 1 month as free-floating cultures in advanced DMEM/F12, 2% B27 minus vitamin A, 2mM L-glutamine, 1% NEAA, 1% P/S and 2 μg/ml Heparin (Thermo Fisher Scientific). This expansion media were supplemented with 20 ng/ml FGF2 and 100ng/ml EGF (Peprotech), and changed twice a week. On day 45 glial progenitors were dissociated using accutase and seeded at a density of 4x10^4^ cells / cm^2^, in flasks coated with 20 μg/ml poly-ornithine and 10 μg/ml Laminin. Plated cells were passaged weekly and grown in expansion media supplemented with 1% FBS allowing spontaneous maturation of glial progenitors into astrocytes, over time. On day 70, astrocyte cultures were fixed and immunocytochemistry was performed for GFAP. The generation of oligodendrocytes was carried out as previously reported ^10^.

*Immunostaining and image acquisition*

Cultures were fixed with 4% paraformaldehyde for 30 min at 4°C, rinsed three times with calcium-free and magnesium-free PBS (PBS-/-) and blocked for 1hr at room temperature with 10% donkey serum in PBS-/- with 0.1% Triton-X (Sigma-Aldrich). Cultures were incubated overnight at 4°C with primary antibodies. The antibodies used were mouse anti-AFP (Sigma-Aldrich; cat# A8452; c=1:500); goat anti-FOXA2 (Santa Cruz; cat# sc-6554; c=1:250), Rabbit anti-LMX1A (Abcam, cat# ab139726; 1:200), mouse anti-SMA (Sigma-Aldrich; cat# A2547; 1:500), rabbit anti-TUJ1 (Covance, cat# PRB-435P; c=1:500), mouse anti-TH (Millipore; cat# MAB318, c=1:2000), and rabbit anti-GFAP (DAKO, cat# Z033401-2, c=1:5000). After three rinses with PBS-/-, appropriate AlexaFluor-488, AlexaFluor-555 and AlexaFluor-647-labelled secondary antibodies (Thermo Fisher Scientific) were used at 1:400 in PBS-/- with 0.1% Triton-X and incubated for 1 hr at room temperature in the dark. DAPI (Life Technologies) was used to counterstain nuclei (1:50 000). Images were acquired using an inverted epifluorescence microscope LRI - Olympus IX-73.

*RT-qPCR for SNCA expression*

RNA extraction was performed using Trizol, and RNA purification through columns according to manufacturer’s instructions (kits ENZA total RNA Kit I: VWR, R6834-02). The Super Script III reverse transcriptase kit was used for reverse transcription experiments (Invitrogen; Cat n: 18080). cDNA was processed using a protocol customized according to the instructions of the SsoFast™ EvaGreen® Supermix (Bio-Rad) in the Bio-rad CFX-96 equipment. Relative quantification was applied using GAPDH as the reference gene. Primers for *hSNCA* and *hGAPDH* were previously reported ^10^.

The expression level of reprogramming factors in hiPSC lines was quantified at approximately passage 13 and compared with non-transduced fibroblasts and a human embryonic stem cell line (H13). Relative quantification was applied using GAPDH as the reference gene. The list of primers used is listed in the table below.

| Primer | Direction | Sequence | Tm (°C) | Source |
| --- | --- | --- | --- | --- |
| pMX | Reverse (R) | TATCGTCGACCACTGTGCTG | 59,4 | This report |
| hKLF4 viral | Forward (F) | CCGCTCCATTACCAAGAGCT | 59,4 | This report |
| hKLF endo | F | GACCACCTCGCCTTACACAT | 58 | Saulnier 2011 |
|  | R | GTTGGGAACTTGACCATGATTG | 58 |  |
| hSOX2 viral | F | GCCCTGCAGTACAACTCCAT | 59,4 | This report |
| hSOX2 endo | F | ACACTGCCCCTCTCACACAT | 58 | Ishikawa 2012 |
|  | R | GGGTTTTCTCCATGCTGTTTCT | 58 |  |
| hcMYC viral | F | CCACTGGTCCTCAAGAGGTG | 61,4 | Takahashi 2007 |
| hcMYC endo | F | AGCAGAGGAGCAAAAGCTCATT | 58 | Mazan-Mamczarz 2006 |
|  | R | CCAAAGTCCAATTTGAGGCAGT | 58 |  |
| pMX R | R | CCCTTTTTCTGGAGACTAAATAAA | 55,9 | Takahashi 2007 |
| hOCT3/4 viral | F | CCCCAGGGCCCCATTTTGGTACC | 67,8 |  |
| hOCT3/4 endo | F | AGTGAGAGGCAACCTGGAGA | 59,4 | Steiner 2010 |
|  | R | GTGAAGTGAGGGCTCCCATA | 59,4 |  |

*Telomerase activity TRAP assay*

A telomeric repeat amplification protocol (TRAP) was employed (telomerase activity assay, TRAPeze, S7700, Millipore) to validate telomerase activity of the reprogrammed iPSCs. In short, total protein was extracted from each of the generated iPSC clones. Selected parental fibroblast lines and/or heat-inactivated iPSC samples were included in each assay as internal negative controls. During an initial 30 min, 30°C incubation, active telomerase in the samples was allowed to add a number of telomeric repeats (GGTTAG) onto the assay-provided substrate oligonucleotide. We then amplified the extended products by PCR and separated the ladder of DNA products by PAGE using 10% TBE gels in 0.5X TBE buffer (Thermo Fisher Scientific) run at 200V for 1 hr. The ladder of products with six base increments started at 50 nucleotides and each sample contained a 36 bp internal standard.

*Karyotyping*

The G-banding analysis was performed in a clinical diagnostic setting. 15-25 metaphasis were analyzed according to the ISCN 2013 ^12^.

*Culture and differentiation of human ventral mesencephalic (VM) precursors*

Human embryonic (5 weeks p.c.) VM precursor cells (procured with approval of the ethics committee of the Medical Faculty of the University of Bern, CH, and the ethics committee of the state of Bern; CH; No. 52/91, 71/94, and 188/95) were isolated and propagated as neural tissue-spheres (NTS) in serum-free culture medium with 20 ng/ml EGF (R&D Systems) and 20 ng/ml FGF2 (R&D Systems), as described elsewhere ^11^. Prior to differentiation, NTS were incubated with trypsin-EDTA (Gibco) diluted 1:10 in D-PBS for 3-5 min at 36°C, dissociated using Pasteur pipettes, centrifuged for 5 min at 4°C (130 x g), and resuspended and plated in poly L-lysine (Sigma-Aldrich) coated 12-well culture trays (Nunc) at a density of 5000 cells/cm^2^. For differentiation, cells were cultured in medium composed of DMEM/F12 w. Glutamax (Gibco), 2% (v/v) 30% glucose (Sigma-Aldrich), 0.5% (v/v) 1M Hepes (Gibco), 2.5% (v/v) AlbuMAX-I (Gibco), 1% (v/v) N2 supplement (Gibco), 1% (v/v) NEAA (Sigma-Aldrich) and 1% penicillin/streptomycin (Gibco) supplemented with 50 ng/ml FGF8 (R&D Systems) for the first three days and then in medium supplemented with 25 μM Forskolin (Sigma-Aldrich), 5 ng/ml GDNF (Promega) and 25 ng/ml SHH (R&D Systems) for the following 7 days before fixation. Cells were differentiated at 36˚C and 3% oxygen tension, with half of the medium being changed every third day. Cultures were fixed in 4% paraformaldehyde in 0.15 M phosphate buffer, pH 7.4 for 20 min, washed in 0.05 M Tris buffered saline (TBS, pH 7.4)/0.1% Triton X-100 (Sigma-Aldrich) and then pre-incubated for 30 min in 0.05 M TBS/10% FBS (Gibco) before incubation with anti-tyrosine hydroxylase (TH, polyclonal rabbit; Chemicon; 1:1200) and anti-glial fibrillary acidic protein antibody (GFAP, monoclonal mouse; Millipore; 1:800) in 0.05 M TBS/10% FBS for 24 hrs. at 4°C. Cells were then rinsed in TBS/0.1% Triton-X-100 and incubated with a mixture of Alexa Fluor® 555 conjugated anti-mouse IgG and Alexa Fluor® 488 conjugated anti-rabbit IgG at 1:200 for two hrs, at room temperature. Cell nuclei were counterstained with DAPI (Sigma-Aldrich). Cultures were mounted using Prolong® Gold mounting medium (Molecular Probes), and images were recorded using a Zeiss Axiophot microscope connected to a Leica DC300 camera.

**References**

1 Liang, G., Taranova, O., Xia, K. & Zhang, Y. Butyrate promotes induced pluripotent stem cell generation. *The Journal of biological chemistry* **285**, 25516-25521, doi:10.1074/jbc.M110.142059 (2010).

2 Unger, C., Skottman, H., Blomberg, P., Dilber, M. S. & Hovatta, O. Good manufacturing practice and clinical-grade human embryonic stem cell lines. *Human molecular genetics* **17**, R48-53, doi:10.1093/hmg/ddn079 (2008).

3 Puttonen, K. A. *et al.* Improved method of producing human neural progenitor cells of high purity and in large quantities from pluripotent stem cells for transplantation studies. *Cell transplantation* **22**, 1753-1766, doi:10.3727/096368912X658764 (2013).

4 Kriks, S. *et al.* Dopamine neurons derived from human ES cells efficiently engraft in animal models of Parkinson's disease. *Nature* **480**, 547-551, doi:10.1038/nature10648 (2011).

5 Xi, J. *et al.* Specification of midbrain dopamine neurons from primate pluripotent stem cells. *Stem cells* **30**, 1655-1663, doi:10.1002/stem.1152 (2012).

6 Krencik, R. & Zhang, S. C. Directed differentiation of functional astroglial subtypes from human pluripotent stem cells. *Nature protocols* **6**, 1710-1717, doi:10.1038/nprot.2011.405 (2011).

7 Hughes, A. J., Daniel, S. E., Kilford, L. & Lees, A. J. Accuracy of clinical diagnosis of idiopathic Parkinson's disease: a clinico-pathological study of 100 cases. *Journal of neurology, neurosurgery, and psychiatry* **55**, 181-184 (1992).

8 Litvan, I. *et al.* Movement Disorders Society Scientific Issues Committee report: SIC Task Force appraisal of clinical diagnostic criteria for Parkinsonian disorders. *Movement disorders : official journal of the Movement Disorder Society* **18**, 467-486, doi:10.1002/mds.10459 (2003).

9 Boulting, G. L. *et al.* A functionally characterized test set of human induced pluripotent stem cells. *Nature biotechnology* **29**, 279-286, doi:10.1038/nbt.1783 (2011).

10 Djelloul, M. *et al.* Alpha-Synuclein Expression in the Oligodendrocyte Lineage: an In Vitro and In Vivo Study Using Rodent and Human Models. *Stem cell reports* **5**, 174-184, doi:10.1016/j.stemcr.2015.07.002 (2015).

11 Andersen, R. K., Widmer, H. R., Zimmer, J., Wahlberg, L. U. & Meyer, M. Leukemia inhibitory factor favours neurogenic differentiation of long-term propagated human midbrain precursor cells. *Neuroscience letters* **464**, 203-208, doi:10.1016/j.neulet.2009.08.050 (2009).

12 Shaffer LG, McGowan-Jordan J, Schmid M. An international system for human cytogenic nomenclature. (Eds); S. Karger, Basel (2013).

**Supplementary figure legends**

*Figure S1: Endogenous and viral expression of pluripotent genes*

1. Up-regulation of endogenous *OCT4, NANOG, LIN28, SOX2, KLF4* and *cMYC* in iPSC lines and human embryonic line (H306) detected by quantitative real time PCR. Values are normalized to house keeping gene *β-ACTIN*.
2. Level of expression of mouse *Oct4*, *Sox2, Klf4* and *cMyc* in UEF-1A line generated using lentivirus containing mouse genes normalized to βactin and compared to expression levels in mouse iPSC line. The detection of the integrated /endogenous genes from UEF-3A and UEF-5G lines generated by using lentivirus containing human “Yamanaka” factors and detected by PCR. Level of expression of viral *OCT4*, *SOX2,* *KLF4* and *cMYC* detected by RT-PCR. Values are compared to house keeping gene GAPDH.

*Figure S2: Endogenous and viral expression of pluripotent genes*

1. Up-regulation of endogenous *KLF4*, *c-MYC*, *OCT4* and *SOX2* in iPSC lines revealed by quantitative real time PCR. Values are normalized to house keeping gene GAPDH and calibrated to marker expression in hESC line H13.
2. Level of expression of viral *KLF4*, *cMYC*, *OCT4*, and *SOX2* in iPSC lines generated using retroviral expression of “Yamanaka” factors. Line CSC-7B has a strong expression of viral *OCT4*. Data are presented as a fold-change to HT1080 human cells overexpressing the 4 factors 3 days following transduction with retroviruses. Values are normalized to house keeping gene GAPDH. The specificity of the primers is confirmed by the lack of viral pluripotent gene expression in both hESC line H13 and parent fibroblasts (ND27760) used to generate lines CSC-3A, -3B, -3G and -3G.
3. Level of expression of viral *KLF4*, *cMYC*, *OCT4*, and *SOX2* in iPSC lines CSC-3S, -7B, 8S, and 9A generated using retroviral expression of “Yamanaka” factors. Data are presented as fold change to endogenous expression of *KLF4*, *cMYC*, *OCT4*, and *SOX2.* Values are normalized to house keeping gene GAPDH.
