# Supplementary figures and images for: Creation of a library of induced pluripotent stem cells from Parkinsonian patients
Source: NPJ Parkinsons Dis. 2016 Jun 2;2:16009–. doi: 10.1038/npjparkd.2016.9 (PMC5516589; doi:10.1038/npjparkd.2016.9)

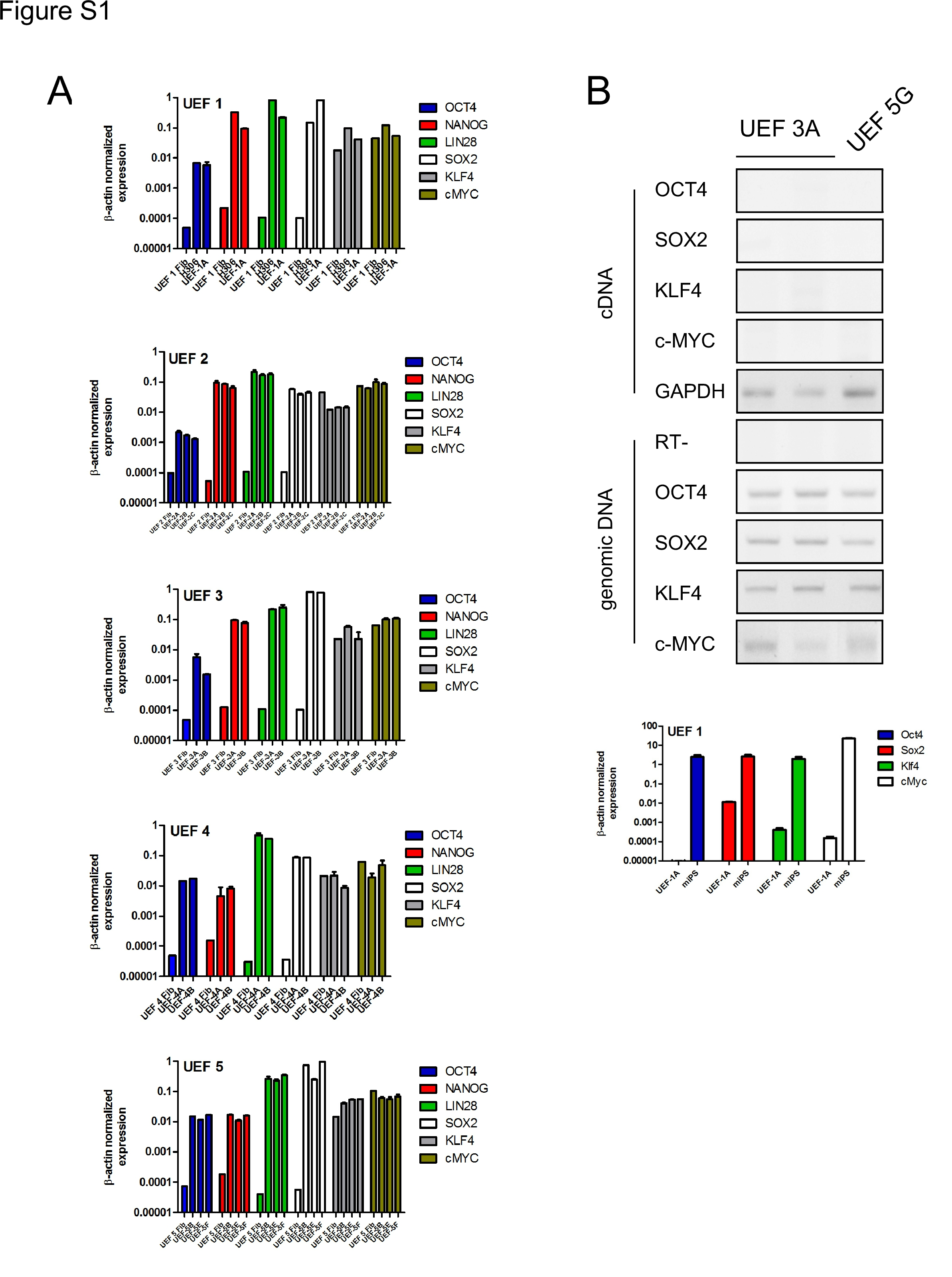

Supplement: Supplementary Figure S1 [file npjparkd20169-s2.jpg]

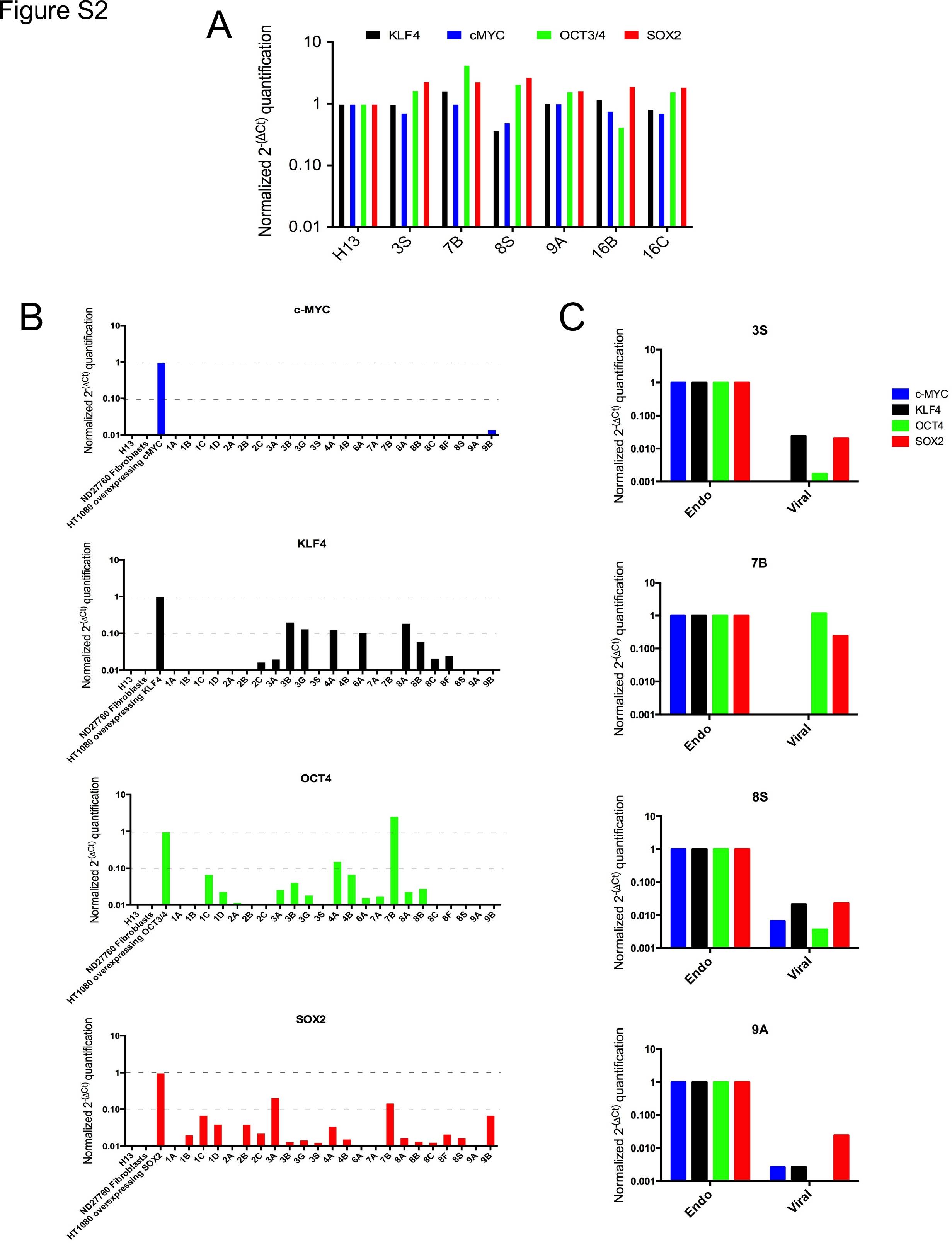

Supplement: Supplementary Figure S2 [file npjparkd20169-s3.jpg]
